# Isocitrate dehydrogenase 1 sustains a hybrid cytoplasmic–mitochondrial tricarboxylic acid cycle that can be targeted for therapeutic purposes in prostate cancer

**DOI:** 10.1002/1878-0261.13441

**Published:** 2023-07-19

**Authors:** Kevin Gonthier, Cindy Weidmann, Line Berthiaume, Cynthia Jobin, Aurélie Lacouture, Camille Lafront, Mario Harvey, Bertrand Neveu, Jérémy Loehr, Alain Bergeron, Yves Fradet, Louis Lacombe, Julie Riopel, Éva Latulippe, Chantal Atallah, Michael Shum, Jean‐Philippe Lambert, Frédéric Pouliot, Martin Pelletier, Étienne Audet‐Walsh

**Affiliations:** ^1^ Endocrinology – Nephrology Research Axis CHU de Québec‐Université Laval Research Center Canada; ^2^ Department of Molecular Medicine, Faculty of Medicine Université Laval Québec Canada; ^3^ Centre de recherche sur le cancer de l'Université Laval Québec Canada; ^4^ Oncology Axis Centre de recherche du CHU de Québec – Université Laval Canada; ^5^ Department of Surgery, Faculty of Medicine Université Laval Québec Canada; ^6^ Anatomopathology Service, Department of Laboratory Medicine CHU de Québec – Université Laval Canada; ^7^ Department of Pathology CHU de Québec – Université Laval Canada; ^8^ Big Data Research Center Université Laval Québec QC Canada; ^9^ Infectious and Immune Disease Axis CHU de Québec‐Université Laval Research Center Canada; ^10^ ARThrite Research Center Université Laval Québec QC Canada; ^11^ Department of Microbiology‐Infectious Diseases and Immunology, Faculty of Medicine Université Laval Québec QC Canada

**Keywords:** androgen receptor, castration‐resistant prostate cancer, citric acid, IDH1, mitochondria, nuclear receptor

## Abstract

The androgen receptor (AR) is an established orchestrator of cell metabolism in prostate cancer (PCa), notably by inducing an oxidative mitochondrial program. Intriguingly, AR regulates cytoplasmic isocitrate dehydrogenase 1 (IDH1), but not its mitochondrial counterparts IDH2 and IDH3. Here, we aimed to understand the functional role of IDH1 in PCa. Mouse models, *in vitro* human PCa cell lines, and human patient‐derived organoids (PDOs) were used to study the expression and activity of IDH enzymes in the normal prostate and PCa. Genetic and pharmacological inhibition of IDH1 was then combined with extracellular flux analyses and gas chromatography–mass spectrometry for metabolomic analyses and cancer cell proliferation *in vitro* and *in vivo*. In PCa cells, more than 90% of the total IDH activity is mediated through IDH1 rather than its mitochondrial counterparts. This profile seems to originate from the specialized prostate metabolic program, as observed using mouse prostate and PDOs. Pharmacological and genetic inhibition of IDH1 impaired mitochondrial respiration, suggesting that this cytoplasmic enzyme contributes to the mitochondrial tricarboxylic acid cycle (TCA) in PCa. Mass spectrometry‐based metabolomics confirmed this hypothesis, showing that inhibition of IDH1 impairs carbon flux into the TCA cycle. Consequently, inhibition of IDH1 decreased PCa cell proliferation *in vitro* and *in vivo*. These results demonstrate that PCa cells have a hybrid cytoplasmic–mitochondrial TCA cycle that depends on IDH1. This metabolic enzyme represents a metabolic vulnerability of PCa cells and a potential new therapeutic target.

## Graphical abstract

The prostate exhibits a unique truncated mitochondrial tricarboxylic acid (TCA) cycle to produce and secrete citrate. Here, we show that during carcinogenesis, prostate cancer cells develop a hybrid cytoplasmic–mitochondrial TCA cycle to reactivate the TCA cycle. This cycle notably depends on the cytoplasmic enzyme isocitrate dehydrogenase 1, which represents a metabolic vulnerability that could be targeted for therapeutic purposes.

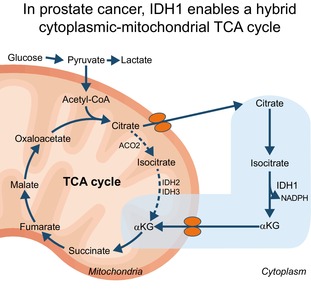

**Abbreviations**
2‐HG: R‐2‐hydroxyglutarate
ACN: acetonitrile
ACO: aconitase
AML: acute myelogenous leukaemia
AR: androgen receptor
CRPC: castration‐resistant PCa
CSS: charcoal‐stripped serum
ECAR: extracellular acidification rates
EI: electron impact ionization
GC–MS: gas chromatography–mass spectrometry
IDH: isocitrate dehydrogenase
MPC2: mitochondrial pyruvate carrier 2
MSKCC: Memorial Sloan‐Kettering Cancer Center
NTC: Non‐Target Control
OCR: oxygen consumption rate
PCa: prostate cancer
PDOs: patient‐derived organoids
PGC‐1α: peroxisome proliferator‐activated receptor‐gamma coactivator‐1 alpha
TCA: tricarboxylic acid cycle
TRC: The RNAi Consortium
αKG: α‐ketoglutarate

## 1 Introduction

The prostate exhibits a unique metabolic program adapted to its secretory functions. Indeed, luminal epithelial cells of the prostate can produce and secrete massive amounts of citrate through a truncated tricarboxylic acid cycle (TCA) [1, 2, 3, 4, 5]. It is believed that the high zinc concentrations in the prostate inhibit the mitochondrial aconitase enzyme (ACO2), thus impairing citrate usage to favour its accumulation for secretion [1, 2, 3, 4, 5]. One important feature during prostate carcinogenesis is the reprogramming of citrate metabolism, leading to a > 10‐ to 100‐fold decrease in zinc and citrate levels in tumours compared to peri‐tumour tissues [1, 6, 7, 8]. Loss of the citrate phenotype occurs in virtually all prostate cancer (PCa) cases, most likely via reprogramming metabolic pathways that consume citrate and by increasing its usage for mitochondrial respiration. Several groups have shown that the androgen receptor (AR), an important oncogenic driver of PCa, promotes mitochondrial respiration in these cancer cells to sustain their aberrant proliferation [4, 9, 10, 11, 12, 13, 14, 15]. The reprogramming of citrate metabolism thus appears to be a PCa hallmark and an interesting new therapeutic target.

The current paradigm is that a “canonical” TCA cycle is re‐established during prostate carcinogenesis, but it has yet to be experimentally demonstrated. It is now well‐known that zinc levels decrease during carcinogenesis, which is believed to enable citrate usage in the mitochondria through the reactivation of ACO2 [15, 16, 17, 18]. In line with this idea, high mitochondrial respiration has recently been evidenced in numerous PCa models, which supports the idea that a canonical TCA cycle is re‐established [10, 11, 12, 14, 15, 19, 20, 21]. However, enhanced oxygen consumption *per se* does not demonstrate restoration of a canonical TCA cycle. In accordance, detailed respiratory assays of mitochondria isolated from PCa tumours have shown a metabolic shift towards higher succinate oxidation [22], indicating a respiratory profile that bypasses ACO2 as well as the downstream mitochondrial isocitrate dehydrogenases (IDHs).

The human genome encodes three IDH isoforms: cytoplasmic IDH1 and mitochondrial IDH2 and IDH3, the latter being a heterocomplex of IDH3A, IDH3B, and IDH3G. IDH1 and IDH2 require NADP^+^ to convert isocitrate into α‐ketoglutarate (αKG), while the IDH3 complex requires NAD^+^ to catalyze its action and to sustain oxidative phosphorylation (respiration). It was previously shown that AR specifically induces IDH1 expression and activity, but not the other IDHs, favouring cytoplasmic over mitochondrial IDH activity [13]. However, the implication of this IDH switch on mitochondrial activity and how IDH1 specifically contributes to citrate metabolism in PCa remains elusive.

Herein, we hypothesized that IDH1 is a critical component of the AR‐driven PCa cell metabolic reprogramming required to maximize mitochondrial respiration by restoring a non‐canonical TCA cycle. We show that pharmacological and genetic blockade of this cytoplasmic enzyme disrupts the mitochondrial capacity of PCa cells, demonstrating that they operate through a hybrid cytoplasmic–mitochondrial TCA cycle. Finally, we provide evidence that this hybrid TCA cycle can be targeted for therapeutic purposes using available inhibitors, including an FDA‐approved molecule, and identify IDH1 as a PCa metabolic vulnerability.

## 2 Materials and methods

### 2.1 Cell culture

LNCaP (RRID: CVCL_1379), LAPC4 (RRID: CVCL_4744), 22Rv1 (RRID: CVCL_1045), DU145 (RRID: CVCL_0105), and PC3 (RRID: CVCL_0035) cells were grown in RPMI1640 medium and VCaP (RRID: CVCL_2235) cells were grown in DMEM (Wisent, Saint‐Jean‐Baptiste, QC, Canada). All cell lines were originally obtained from the ATCC; they were all authenticated in 2017 by the ATCC (Manassas, VA, USA), and frozen stock of no more than 3 years from this authentication were used for resuscitation to perform the current study. All experiments were performed with mycoplasma‐free cells, with detection tests performed every 3–4 months. Media were supplemented with 10% FBS (Wisent), 100 U·mL^−1^ penicillin–100 μg·mL^−1^ streptomycin (Wisent), and 1 mm sodium pyruvate (Thermo Fisher Scientific, Montréal, QC, Canada) at 37 °C and 5% CO_2_. Hormonal treatments were performed with the synthetic androgen R1881 (10 nm) (Steraloids, Newport, RI, USA) or estradiol (Sigma, St. Louis, MO, USA) (E_2_; 10 nm) following a 48 h steroid deprivation in phenol red‐free RPMI1640 (Wisent) or DMEM (Wisent) with 5% charcoal‐stripped serum (CSS) as described previously [12, 23]. Knockdown by siRNA was performed by transfecting PCa cells with IDH1‐targeting siRNA or a non‐targeted control (Dharmacon, Lafayette, CO, USA) using the Hiperfect reagent according to the manufacturer's instructions (Qiagen, Toronto, ON, Canada) 1 h post‐plating in media with 5% CSS, as previously described [13]. IDH1 pharmacological inhibition was achieved by treating cells with 5 μm of GSK321 (Medkoo Bioscience, Morrisville, NC, USA) or AG‐120 (Medkoo Bioscience).

### 2.2 Animal studies

All animal experimental protocols involved male mice and were approved by the Animal Research and Ethics Committee of Université Laval (Project 22‐1206). All methods involving mouse work were carried out in accordance with Université Laval's Animal Research and Ethics Committee guidelines and regulations. Mice were bred, housed in a 12 h light:12 h dark cycle at 22 °C, and handled at the animal care facility of the CHU de Québec‐Université Laval Research Center. For xenograft experiments, 22Rv1 cells were first trypsinized, counted, and resuspended at a concentration of 7.5 million·mL^−1^ in a 50% (Wisent) PBS–50% Matrigel (Fisher Scientific) solution. Each mouse (strain: Crl:CD1‐Foxn1nu – Charles River) (~ 70 days old) received a subcutaneous injection of 200 μL (1.5 million cells) in the right flank. Tumour growth was evaluated twice a week using calipers. When tumours reached the endpoint, i.e., a tumour volume of 1.5 cm^3^, mice were sacrificed. Tumours were harvested and flash frozen before being stored at −80 °C for downstream analysis.

### 2.3 IDH enzymatic activity

Total and NAD^+^‐specific or NADP^+^‐specific IDH enzymatic assays were performed using the IDH Activity assay kit (Sigma) as previously described [13]. For whole‐cell lysate analyses, cells were collected in ice‐cold PBS. Cells were then centrifuged at 800 ***g*** for 5 min at 4 °C and counted using a TC10 automated cell counter (Bio‐Rad, Saint‐Laurent, QC, Canada). Next, cells were resuspended in ice‐cold IDH assay buffer at a concentration of one million cells per 200 μL. Cell lysates were diluted 1 : 10 before determining NADP^+^‐dependent and NAD^+^‐dependent IDH activity using NADP^+^ or NAD^+^ substrate, respectively, by following the manufacturer's instructions. Absorbance measurements of optical density at 450 nm were made using a spectrophotometer. For the tissue‐specific IDH activity assay, three 12–18‐week‐old male wild‐type C57BL/6 mice were sacrificed for each experiment. Organs were harvested in ice‐cold PBS before being homogenized with microtube pestles in ice‐cold IDH assay buffer. Tissue homogenates were centrifuged at 10 000 ***g*** for 10 min, and supernatants were used for the assay. For each sample, 20 μL of lysates (normalized for protein content) were mixed with 20 μL of reaction mix that was prepared according to the manufacturer's guidelines.

### 2.4 Cell fractionation

For cell line experiments, cells were first passaged and then 5 million cells/10 cm tissue culture dish were plated. After 24 h, cells were washed with ice‐cold PBS, harvested using a cell scraper, and centrifugated at 2 000 ***g*** for 5 min at 4 °C. Pellets were resuspended in Mitochondrial Isolation Buffer (250 mm sucrose, 5 mm HEPES, 2 mm EGTA, ddH_2_O pH 7.2) (MIB) [24] before being homogenized using a Dounce homogenizer. Lysates were collected and centrifuged at 900 ***g*** for 10 min at 4 °C. Supernatants were again centrifuged at 900 ***g*** for 10 min at 4 °C and then underwent high‐speed centrifugation (10 000 ***g*** × 10 min at 4 °C). Supernatants (cytoplasm) were collected and kept on ice. Pellets (mitochondria) were washed and centrifuged at 10 000 ***g*** × 10 min at 4 °C. Purified cytoplasmic and mitochondrial extracts were then used to perform IDH activity assays using NAD^+^ and/or NADP^+^ as co‐substrates. Data were normalized per microgram of protein and for total lysate (to adjust for the cytoplasmic and mitochondrial fractions ratio). The same protocol was used for tissue enzymatic analyses. Cytoplasmic and mitochondrial fraction purities were verified by western blots.

### 2.5 RNA extraction and quantitative reverse transcription PCR

RNA was isolated and purified using the RNeasy purification kit (Qiagen). cDNA was synthesized using the LunaScript RT SuperMix Kit from New England BioLabs (NEB, Ipswich, MA, USA). Gene expression was measured by quantitative PCR using the Luna Universal qPCR Master Mix (NEB) in technical duplicates for each sample. Relative expression of human target genes was obtained by normalization against the expression of the housekeeping genes *TBP* and *PUM1*. Primer sequences are detailed in Table S1.

### 2.6 Western blot

Protein extraction from cells was performed using buffer K supplemented with protease inhibitor cocktail (MilliporeSigma, Oakville, ON, Canada) and PhosSTOP™ phosphatase inhibitors (MilliporeSigma) as described previously [12]. Relative levels of proteins were measured with imagej software and normalized over tubulin. Results are shown as the average and SEM of at least three independent experiments. The primary antibodies used were from Abcam (Toronto, ON, Canada): IDH1 (ab172964); Cell Signaling (Danvers, MA, USA): IDH1 (8137), IDH2 (56439S), Citrate Synthase (14309), Tubulin (2125); and Santa Cruz Biotechnology (Santa Cruz, CA, USA): AR (sc‐7305), IDH3A (sc‐398021), S6 (sc‐74459), P‐S6 (sc‐293144).

### 2.7 Extracellular flux analyses

Oxygen consumption rates (OCR) and extracellular acidification rates (ECAR) were measured using the extracellular flux analyser XFe96 from Agilent Seahorse Bioscience as previously optimized for PCa cells [10, 12, 19]. Cells were plated at 10 000 cells/well confluency in a 96‐well Seahorse microplate in phenol red‐free RPMI medium supplemented with 5% CSS. After 48 h, cells were treated with androgens and/or IDH1 inhibitors. After another 48 h, media was changed for Seahorse XF RPMI Medium supplemented with 10 mm glucose, 2 mm glutamine, 1 mm sodium pyruvate, and 100 U·mL^−1^ penicillin–100 μg·mL^−1^ streptomycin. The microplate was then transferred to a CO_2_‐free incubator at 37 °C for 1 h. After equilibration, the microplate was inserted into the XFe96 instrument. Basal respiration as well as respiration during a mitochondrial stress test were measured by three consecutive measurements of OCR and ECAR before and after injection of oligomycin (Sigma), FCCP (Santa Cruz), and antimycin A (Sigma) + rotenone (Sigma). For normalization, cell counting was performed using CyQUANT (Thermo Fisher Scientific) assay as described previously [25].

### 2.8 Human primary prostate cell culture conditions

The human experiments were undertaken with the understanding and written consent of each subject following approval by the research ethics committee of the CHU de Québec – Université Laval (Project 2021‐5661). The study methodologies conformed to the standards set by the Declaration of Helsinki. The human prostate organoids were generated as previously described [5], with all samples being obtained at the Hôtel‐Dieu de Québec between March 2020 and March 2022. In brief, normal and tumour prostate tissues were harvested from freshly resected radical prostatectomy samples under direct supervision of a pathologist. Samples were respectively harvested in regions without and with indication of PCa and later confirmed at the pathology department. After harvest, tissues were enzymatically digested to obtain a single‐cell suspension. Following digestion, cells were plated for 2‐dimensional cell culture for initial amplification in optimized KSFM media [5]. TrypLE (GIBCO, Stratford, ON, Canada) supplemented with 10 μm Y‐27632 was used to pass the cells (with no more than 5 passages in primary culture). For 3‐dimensional culture, cells were passaged and resuspended in complete human organoid medium, as optimized previously [5]. Matrigel was then added to plate 40 μL droplets containing 75% of growth factor‐reduced Matrigel and 15 000 cells/droplet. Fifteen minutes after plating and incubation at 37 °C, warm complete organoid media were added to each well. The media were renewed twice a week for the complete duration of the experiments. Normal patients CW488 (PDO_#1) and CW513 (PDO_#2) were used in the current study and the tumour sample CW513.

### 2.9 Histology and microscopy analyses

For fixation, organoids‐containing matrigel droplets were first rinsed in PBS (1 mL/well), removed from wells, and then transferred to 1.5 mL micro‐centrifuge tubes before being centrifuged (2500 ***g*** × 2 min at 4 °C). After discarding the supernatant, pellets were resuspended in PBS (1.5 mL) and underwent another centrifugation (2500 ***g*** × 2 min at 4 °C). Afterwards, a 4% paraformaldehyde solution was added to the matrigel droplets and incubated at room temperature. After 1 h, droplets were again centrifuged (2500 ***g*** × 3 min at 4 °C) and supernatants were discarded. A 3% agarose solution was added to the matrigel droplets for fixation, which were then moved into histological cassettes and incubated in 10% formalin overnight at room temperature. Another fixation of the droplets was then performed in paraffin (Laboratoire de pathologie de l'Hôtel‐Dieu de Québec). Finally, hematoxylin and eosin (H&E) staining was performed on 5 μm fixed droplets obtained using a microtome (HistoCore MULTICUT 14051856372, Leica). Acquisition of images was performed with an EVOS™ M5000 Imaging System (Thermo Fisher Scientific).

### 2.10 shRNA‐mediated knockdown

Sequences of shRNAs targeting IDH1 (termed shIDH1_1 and shIDH1_2) were selected from The RNAi Consortium (TRC) whole‐genome library. The shRNA Non‐Target Control (NTC) sequence was the one published by Lu et al. [26]. Using these sequences detailed in Table S2, each shRNA was cloned in the EZ‐Tet‐pLKO‐Puro vector (Addgene plasmid # 85966; Watertown, MA, USA), as described by Frank et al. [27]. Once cloned, these vectors were used in co‐transfection with plasmids psPAX2 and pMD2.G into Lenti‐X HEK293T cells for lentiviral production. Two days following transfection, the supernatants were filtered with a 0.45 μm filter. Human cells were then transduced with viruses‐containing supernatant. For LNCaP cells, a ratio of 1 : 10 of supernatant was added to the culture media. After 48 h, 10 ng·mL^−1^ of doxycycline was added to induce the shRNA expression. After 7 days of treatment, cells were harvested for experiments. After passage, human primary prostate epithelial cells were directly resuspended in the supernatant containing the viruses. After 30 min, Matrigel was added to perform organoid culture following the standard protocol described above. Doxycycline at 100 ng·mL^−1^ was added after 4 days of culture until complete maturation of the organoids, i.e., 14 days.

### 2.11 Cell proliferation

Cells were plated (5000–10 000 cells/well) in 96‐well plates in appropriate media with 5% CSS. After 48 h of steroid deprivation, cells were treated with hormones, vehicles, or IDH1 inhibitors for the indicated duration. At the end of the experiment, media was removed and 50 μL/well of a 0.5% crystal violet staining solution was added to perform cell counting for LNCaP, 22Rv1, LAPC4, VCaP, 22Rv1 control, and 22Rv1 IDH1‐KO cells. Plates were incubated for 30 min at room temperature on a bench rocker after which cells were washed four times with water and dried at room temperature overnight, protected from light. Cells were then lysed in 200 μL/well of 2% SDS for 4 h at room temperature. A spectrophotometer was used to measure optical density (OD) at 570 nm. Standard curves for each cell line were used to convert OD values to cell numbers.

### 2.12 *IDH1* knockout

CRISPR‐mediated knockout (KO) of the *IDH1* gene was achieved by designing a guide RNA (gRNA) (guide sequence: GATCCTTGGTGACTTGGTCGT) based on the computation of genomic coordinates of IDH1 exon 4 using the CRISPOR tool [28]. The DNA sequence of this gRNA was then cloned into the eSpCas9_G2 plasmid as previously published [29] (Addgene plasmid # 86612; Watertown, MA, USA). Sequences were validated with Sanger sequencing (forward: TCCCCATAAGCATGACGACC; reverse: CTCCCACCACTTCAACGTCA) at the CHU de Québec‐Université Laval Research Center. The day before transfection, 500 000 22Rv1 cells per well were plated in a six‐well plate. Cells were transfected with 600 ng of the construct using the FuGENE HD Transfection Reagent (Promega, Fitchburg, WI, USA) according to the manufacturer's instructions at a ratio of 0.3 μL Transfection Reagent: 100 ng DNA per well. Seventy‐two hours after transfection, 0.5 μg·mL^−1^ of ouabain was added to the culture media for selection. Single clones were then isolated in 96‐well plates after selection and amplified. Finally, clones were screened, and KO was confirmed by Sanger sequencing and western blots.

### 2.13 Gas chromatography–mass spectrometry

Cells were rinsed with ice‐cold NaCl (0.9%) solution on ice before being scraped with 500 μL of cold 80% MeOH on dry ice and stored at −80 °C. For tumour tissues, tumours were grinded to powder using a mortar and pestle on dry ice and stored at −80 °C. Immediately before extraction, tissue power was resuspended into 500 μL of cold 80% MeOH. For metabolite extraction, cells and tissues were sonicated for 20 min in 30 s intervals in ice‐cold water and then centrifugated at 20 000 ***g*** for 10 min. After being transferred to new tubes, supernatants were vortexed with the internal myristic acid‐d27 (CDN Isotopes, Pointe‐Claire, QC, Canada) and 700 μL ice‐cold acetonitrile (ACN). Samples then underwent nitrogen gas‐based evaporation until complete dryness. For derivatization, methoxiamination of samples was done according to the protocol of Fiehn [30]. Silylation using MTBSTFA–1% TBDMCS (Sigma Aldrich, St.‐Louis, MO, USA; TCI America, Cambridge, MA, USA) was then performed according to the protocol of Patel et al. [31] with slight modifications. Sample analysis by GC–MS was conducted with an Agilent 8890 GC equipped with a DB5‐MS + DG capillary column coupled to an Agilent 5977B MS instrument using 70 eV‐electron impact ionization (EI) (Agilent Technologies, Santa Clara, CA, USA). One microlitre of each sample was injected into the GC at 250 °C in split mode with a helium flow rate of 1 mL·min^−1^ as carrier gas. The GC oven temperature was kept at 50 °C. After 2 min, the temperature was raised at intervals of 20 °C·min^−1^ up to 150 °C. The temperature was then raised by 10 °C·min^−1^ intervals up to 300 °C and was maintained for 10 min. The MS source temperature was maintained at 230 °C and the quadrupole temperature was kept at 150 °C. For detection, the mass range was 50–600 Da at a signal rate of 5.1 scans·s^−1^. The Agilent MassHunter Workstation Software was used for analyses. Identification of metabolites was performed with the NIST/EPA/NIH Mass Spectral Library (NIST 201, Gaithersburg, MD, USA). TCA cycle metabolites were quantified in absolute levels using corresponding standards. For stable isotope tracer analyses, cells were incubated with ^13^C‐labelled glucose (10 mm) for 24 h. Samples were then prepared as described above. After quantification of isotopomers, natural ^13^C abundance was corrected using accucor [32]. Student's *t*‐tests were used for statistical analyses.

### 2.14 Clinical datasets

The correlation analysis data were obtained from the Hutchinson clinical dataset [33] and the Taylor et al. [34] dataset, accessed in November 2021.

## 3 Results

### 3.1 IDH1 is induced by AR in PCa

**Fig. 1:**
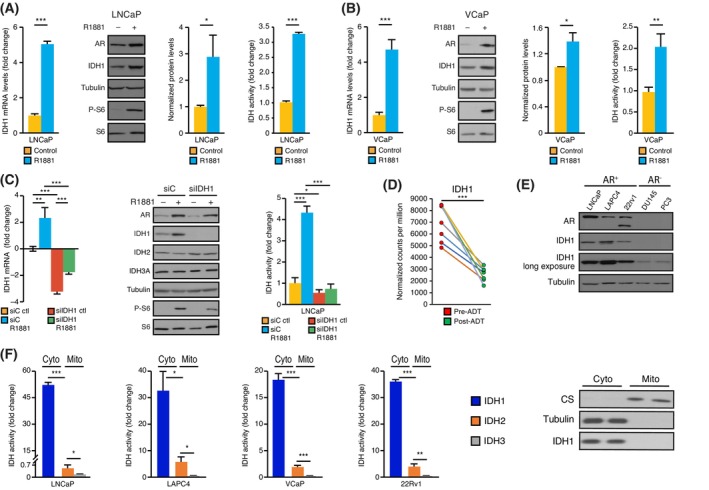
AR controls the expression of IDH1 in PCa. (A) IDH1 mRNA and protein expression levels in LNCaP cells following 48 h of treatment with the synthetic androgen R1881. Tubulin was blotted as a protein loading control, and phosphorylation of S6 (P‐S6) was used as a positive control for AR activation. Densitometric quantification of IDH1 intensity over tubulin intensity is the mean of three independent experiments. Total IDH activity fold change following treatment with R1881 (10 nm) is shown as the mean ± SEM (*n* = 3). (B) IDH1 mRNA and protein expression levels in VCaP cells following 48 h of treatment with R1881. Tubulin was blotted as a protein loading control and P‐S6 was used as a positive control for AR activation. Densitometric quantification of IDH1 intensity over tubulin intensity is shown as the mean of three independent experiments. Total IDH activity fold change following treatment with R1881 is shown as the mean ± SEM (*n* = 3). (C) IDH mRNA and protein expression levels in LNCaP cells transfected or not with scrambled‐siRNA (siC) or IDH1‐targeting siRNA (siIDH1) for 96 h, then treated for 48 h with R1881. Tubulin was blotted as a protein loading control and P‐S6 was used as a positive control for AR activation. Total IDH activity fold change is shown as the mean ± SEM (*n* = 3). (D) *IDH1* expression (Counts Per Million, CPM) from RNA‐seq data in PCa tissues before and after 22 weeks under androgen deprivation therapy (ADT; *n* = 7) from Rajan et al. (GSE48403) [35]. (E) IDH1 protein expression across PCa cell lines (AR^+^: LNCaP, LAPC4, 22Rv1; AR^−^: DU145, PC3). Tubulin was blotted as a protein loading control. One representative experiment is shown out of three independent experiments. (F) Cytoplasmic (Cyto) and mitochondrial (Mito) IDH activities of PCa cells. Western blot for fractionation control is shown for 22Rv1 cells (right) with tubulin and citrate synthase (CS) used respectively as cytoplasmic and mitochondrial controls. For enzymatic assays, results are shown as the mean ± SEM (*n* = 3). Statistics shown used the Student's *t*‐test. *P < 0.05; **P < 0.01; ***P < 0.001.

It was previously shown that AR positively regulates the *IDH1* mRNA levels in human PCa cell lines by binding to the androgen response elements in close vicinity to the *IDH1* promoter [13]. This regulation of *IDH1* mRNA by AR, using the synthetic androgen R1881, was validated herein for LNCaP (Fig. 1A) and VCaP (Fig. 1B) cells. Moreover, IDH1 protein levels were also shown to be increased following androgen stimulation, correlating with an increase in total IDH activity in LNCaP (Fig. 1A), VCaP (Fig. 1B), and 22Rv1 (Fig. S1A) cells. Specific siRNA‐mediated knockdown of *IDH1* significantly decreased its mRNA levels (Fig. 1C). Importantly, *IDH1* knockdown strongly impaired IDH1 protein levels and completely blocked the AR‐dependent induction of IDH1 protein levels and the total IDH activity driven by AR (Fig. 1C), emphasizing that IDH1 is the major contributor to the total IDH activity in PCa cells. In matched PCa tissues collected from patients before and after androgen deprivation therapy [35], *IDH1* was significantly decreased following androgen deprivation (Fig. 1D), validating the positive relationship between *IDH1* gene regulation and AR activity *in vivo* in human PCa cells. In PCa cell lines, AR‐positive (AR^+^) PCa cells displayed strong IDH1 protein expression (Fig. 1E). All AR^+^ PCa cells specifically showed high cytoplasmic IDH1 activity and low mitochondrial IDH activity (Fig. 1F and Fig. S1B). AR‐negative (AR^−^) PCa cell lines (DU145 and PC3) showed three times lower IDH activities compared to AR^+^ PCa cells; however, as AR^+^ PCa cells, they also exhibited an IDH profile relying on NADP^+^ compared to NAD^+^ (Fig. S1B). Overall, these results validate that androgens not only upregulate *IDH1* mRNA expression and activity [13] but also demonstrate regulation of IDH1 protein levels following androgen stimulation. Moreover, the current results also show that AR^+^ PCa cell lines rely more on cytoplasmic IDH1 than on mitochondrial IDHs.

### 3.2 Mouse prostate and human prostate organoids exhibit an IDH1‐dependent metabolic profile

**Fig. 2:**
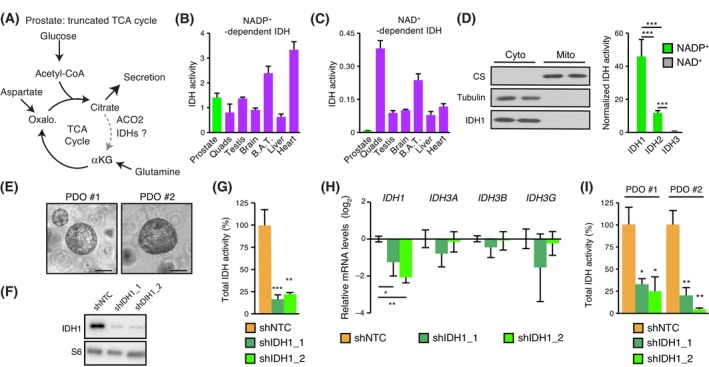
IDH1 is the predominant IDH in the normal prostate. (A) Schematic representation of the TCA cycle that occurs in the prostate. High levels of citrate are secreted due to a truncated TCA cycle. The aconitase 2 enzyme (ACO2) is known to be mostly inactive, while the extent of the mitochondrial IDH activity remains to be determined. Glutamine was recently shown to allow completion of the rest of the cycle by replenishing αKG [5]. Oxalo, oxaloacetate. NADP^+^‐dependent (B) and NAD^+^‐dependent (C) IDH activity in several mouse tissues (B.A.T., Brown adipose tissue; quads, quadriceps). Values are normalized by μg of protein. Results are shown as the mean ± SEM of one independent experiment performed with three animals, out of two independent experiments. (D) Cytoplasmic (Cyto) and mitochondrial (Mito) IDH activities of normal mouse prostate. Citrate synthase (CS) and tubulin were blotted as fractionation controls. Results are shown as the mean ± SEM (*n* = 3); one out of three independent experiments is shown. (E) Brightfield visualization of human normal prostate patient‐derived organoids (PDOs) after 14 days in culture. Scale bars = 100 μm. (F) IDH1 protein expression in LNCaP cells following expression of shIDH1 or a non‐targeted control (NTC). S6 is shown as a protein loading control. (G) Total IDH activity in LNCaP cells after transduction with shRNA (as in F). Results are shown as the mean ± SEM (*n* = 3) of one out of three independent experiments. (H) qRT‐PCR analysis of IDHs genes expression in PDO#2. Cells were transduced before being plated for organoid growth. After 4 days in culture, doxycycline was added to trigger shRNA expression and proper knockdown of *IDH1*. Ten days later, organoids were harvested for analysis. Results are shown as the mean ± SEM (*n* = 3) of one representative experiment. (I) Total IDH activity in two PDOs after transduction with shRNA, as in (H). Results are shown as the mean ± SEM (*n* = 3). Statistics shown used the Student's *t*‐test. *P < 0.05; **P < 0.01; ***P < 0.001.

To better understand the origins of this specific IDH1‐dependent profile of AR^+^ PCa cells, we sought to determine the IDH‐associated metabolic program in the normal prostate. In luminal (AR^+^) epithelial cells, the TCA cycle is truncated at the ACO2 step that is upstream of mitochondrial IDHs (Fig. 2A). Initially believed to be fully truncated between citrate and oxaloacetate, glutamine has been recently shown to fuel the TCA cycle by replenishing αKG, just downstream mitochondrial IDHs [5]. These results thus suggest that mitochondrial IDHs are mostly inactive in the normal prostate. To test this hypothesis, we determined IDH1, IDH2, and IDH3 enzymatic activities across various tissues. Cytoplasmic IDH1 and mitochondrial IDH2 enzymes require NADP^+^, while the mitochondrial IDH3 complex specifically uses NAD^+^. We first measured basal IDH activities consuming NAD^+^ and NADP^+^ in several mouse tissues. Whereas NADP^+^‐specific IDH activity was observed in all tested tissues (Fig. 2B), the prostate showed an absence of or only trace amounts of detectable NAD^+^‐specific IDH activity (Fig. 2C), indicating a lack of or only weak IDH3 activity. Subcellular fractionation was then performed to distinguish between cytoplasmic IDH1 and mitochondrial IDH2 for NADP^+^ utilization, identifying IDH1 as the most active IDH complex in the normal mouse prostate (Fig. 2D). In line with these results, IDH1 protein expression was easily detectable, while IDH2 and IDH3A protein levels were barely detectable (Fig. S2A). Altogether, these results show that, along with the upstream enzyme ACO2 that was shown to be mostly inactive [36], the mitochondrial IDH complexes are also mostly inactive in the normal mouse prostate, with IDH3 activity being barely detectable at all.

Next, we wanted to determine the contribution of IDH1 in a human prostate model. To do so, two normal prostate organoid models were used (Fig. 2E). These were generated from fresh human prostate biopsies taken after radical prostatectomy in zones without any evidence of PCa and as distant as possible from zones with cancer cells, as previously described [5]. *Ex vivo*, human prostate epithelial cells can be grown in 3‐dimensional culture and form organoids that recapitulate the glandular structure of the prostate (Fig. 2E and Fig. S2B). Mitochondrial purification could not be performed in these patient‐derived organoids (PDO) due to limited biological samples. Consequently, we used a genetic approach to determine the contribution of IDH1 to their total IDH activity. As siRNAs are transient by nature and given that prostate organoids must grow 14 days to reach their maximal size, lentiviral transduction of two inducible shRNAs against *IDH1* (termed shIDH1_1 and shIDH1_2) was first optimized in LNCaP cells. These two shIDH1s were shown to be specific to *IDH1*, significantly decreasing its mRNA and protein levels, with no significant impact on other IDH genes (Fig. 2F and Fig. S2C,D). Consequently, transduction with shIDH1_1 and shIDH1_2 led to a significant decrease of > 75% of IDH1 activity in LNCaP cells (Fig. 2G), similar to what we observed using siRNAs (Fig. 1C). After validation, we used these two shIDH1s in the two PDOs, which significantly decreased IDH1 mRNA (Fig. 2H) and protein levels (Fig. S2E) specifically. Knockdown of *IDH1* in both human organoid models led to a significant decrease of 67% to 90% total IDH activity (Fig. 2I), indicating, as for the mouse prostate (Fig. 2B–D) and human PCa models (Fig. 1), a predominant contribution of IDH1 to the cumulative IDH activity profile. Altogether, these results indicate that both mouse and human prostate cells exhibit low mitochondrial IDH2‐IDH3 activities downstream ACO2 and that they instead exhibit high cytoplasmic IDH1 activity. Consistent with the inactivation of mitochondrial ACO2 in the prostate, our results show an inactive IDH3 complex and low activity of IDH2 in the normal prostate.

### 3.3 AR stimulates IDH1 in PCa to support mitochondrial respiration

Based on the results above showing a strong IDH1 reliance in numerous normal prostate models, we hypothesized that PCa exhibits an IDH1‐dependent metabolic profile that originates from the normal prostate metabolic program. In tumours, rather than reactivating IDH2 and IDH3, cancer cells remain dependent on this highly active cytoplasmic IDH1. Yet, what role(s) IDH1 plays following AR activation in PCa cells remains elusive.

**Fig. 3:**
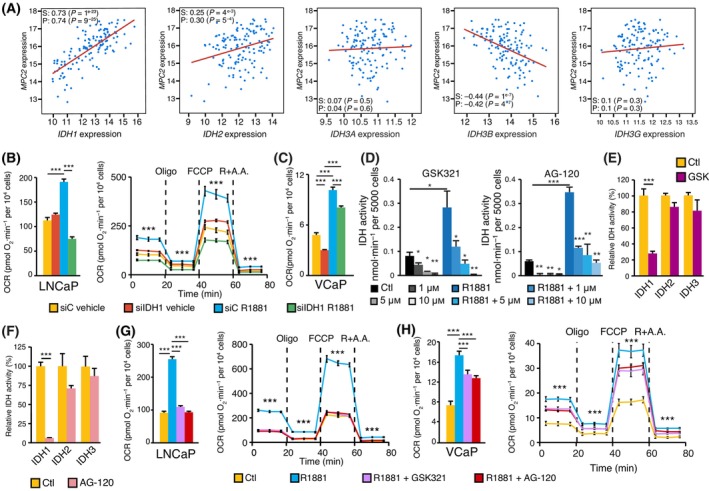
AR‐dependent induction of cytoplasmic IDH1 promotes mitochondrial respiration in PCa. (A) Correlation analysis of *IDH1*, *IDH2, IDH3A*, *IDH3B*, and *IDH3G* mRNAs with *MPC2* mRNA from the Fred Hutchinson CRPC dataset. (B) Basal respiration (left) and the complete mitochondrial stress test (right) are shown for LNCaP cells, with and without treatment with vehicle or the synthetic androgen R1881 for 48 h, and transfected with scrambled‐siRNA (siC) or IDH1‐targeting siRNA (siIDH1). Results are shown as the mean ± SEM (*n* = 10–12). In the complete mitochondrial stress panel, dashed lines indicate the sequential injections of oligomycin (oligo), the uncoupler FCCP, and rotenone + antimycin A (R + A.A.). (C) Basal respiration of VCaP cells treated with vehicle or R1881 (10 nm) for 48 h and transfected with scrambled‐siRNA (siC) or IDH1‐targeting siRNA (siIDH1). Results are shown as the mean ± SEM (*n* = 10–12). (D) Total IDH activity in LNCaP cells untreated or treated with 1, 5, or 10 μm of IDH1 inhibitors GSK321 or AG‐120 for 48 h, with or without R1881 co‐treatment. Results are shown as the mean ± SEM (*n* = 3). Cytoplasmic and mitochondrial IDH activities of LNCaP cells untreated or treated with 5 μm of GSK321 (GSK) (E) or AG‐120 (F) for 48 h (*n* = 3) in complete media (with androgens). The activity in vehicle‐treated cells was set at 100% for each IDH. (G) Basal respiration (left) and the complete mitochondrial stress test (right) are shown for LNCaP cells. Before measurement of OCR, LNCaP cells treated with and without R1881, GSK321, and AG‐120 for 48 h. Results are shown as the mean ± SEM (*n* = 10–12). (H) Basal respiration of VCaP cells (left) and the complete mitochondrial stress test (right) are shown. Before measurement of OCR, VCaP cells were treated with and without R1881, GSK321, and AG‐120 for 48 h. Results are shown as the mean ± SEM (*n* = 10–12). Statistics shown used the Student's *t*‐test. *P < 0.05; **P < 0.01; ***P < 0.001. All the experiments were performed at least three times independently.

Even though it is cytoplasmic, IDH1 catalyzes a reaction often associated with the mitochondrial TCA cycle, and we thus hypothesized that it could be connected to the TCA cycle and mitochondrial respiration. The TCA cycle most often begins with the import of pyruvate to mitochondria to generate acetyl‐CoA, an essential metabolite that provides two‐carbon units to replenish citrate and enable its cycling. The mitochondrial pyruvate carrier 2 (MPC2), which allows mitochondrial import of pyruvate, was recently shown to be required for mitochondrial respiration in PCa cells [14]. It was demonstrated that MPC2 was higher in tumours than in normal tissues and that its expression was driven by AR [14]. We thus explored the relationship between *MPC2* and the different IDHs in PCa using the data from the Fred Hutchinson castration‐resistant PCa (CRPC) study [14, 33]. A significant correlation was observed between *MPC2* and *IDH1* (Spearman correlation coefficient [cc] of 0.74, *P* = 9^−25^; Fig. 3A) in this aggressive PCa setting. *IDH2* was modestly correlated with *MPC2* (cc of 0.30) while genes of the IDH3 subfamily were not correlated or negatively correlated to *MPC2* (Fig. 3A). Validation of these correlations was performed using the independent cohort of the Memorial Sloan‐Kettering Cancer Center (MSKCC) (Fig. S3A) [34]. Notably, highly similar results were obtained, demonstrating a high correlation between *MPC2* and *IDH1* specifically (cc of 0.65, *P* = 4^−16^) and low correlation with other IDHs (cc < 0.25). As PCa cell mitochondrial respiration was shown to be dependent on MPC2 and given the high correlation between *MPC2* and *IDH1*, we hypothesized that IDH1, despite its cytoplasmic localization, could be linked to the MPC2‐dependent mitochondrial respiration.

To test this hypothesis, we measured OCRs of LNCaP and VCaP cells. Following androgen stimulation with R1881, a significant increase in OCR was observed in both cell lines, for basal and maximal respiration (Fig. 3B,C, and Fig. S3B), a well‐appreciated metabolic function of AR [10, 11, 12, 14, 15, 19, 20]. Importantly, this AR‐mediated effect was completely blocked following the knockdown of *IDH1* in LNCaP cells and was significantly decreased in VCaP cells after the knockdown of *IDH1* (Fig. 3B,C, and Fig. S3B). Consequently, these results support the hypothesis that IDH1 is required to support mitochondrial respiration in these PCa cells. Another important ATP‐generating pathway is aerobic glycolysis, which can use pyruvate to generate lactate and ATP in a mitochondrial‐independent manner. Along with measuring OCR, we also measured ECAR, a proxy of aerobic glycolysis, following the knockdown of *IDH1*. For both LNCaP and VCaP cells, we did not observe a switch towards glycolysis, but an impairment of glycolysis following *IDH1* knockdown (Fig. S3C,D).

To validate our results with siRNAs, we sought to use IDH1 inhibitors. Pharmacological inhibitors of mutant IDH1 with validated safety profiles are currently used in the clinic to treat patients with tumours harbouring *IDH1* mutations [37, 38]. Mutation of this gene is indeed observed with high frequency in specific blood and brain cancers [39, 40] but rarely in PCa [41]. The encoded protein has a new enzymatic function that produces the oncometabolite *R*‐2‐hydroxyglutarate (2‐HG) causing oncogenesis [42, 43]. Inhibitors such as GSK321 and AG‐120 were designed to target this mutant IDH1 enzyme with high affinity, but interestingly they can also inhibit wild‐type IDH1 at higher concentrations, with no inhibitory activity against IDH2 [37, 38]. We thus hypothesized that these inhibitors could be repurposed to block wild‐type IDH1 in our models. In PCa cells harbouring wild‐type IDH1, treatment with GSK321 and AG‐120 caused a dose‐dependent decrease in total IDH activity and abolished its induction by AR (Fig. 3D), as was observed after *IDH1* knockdown (Figs 1 and 2). In parallel, IDH1 inhibition with GSK321 did not inhibit the citrate synthase (CS) enzyme (Fig. S3E). To further validate the specificity of this pharmacological strategy, we combined GSK321 and AG‐120 with cell fractionation to delineate the individual IDH activity. Such assay demonstrated that GSK321 and AG‐120 inhibitors are specific to wild‐type IDH1, decreasing >80% of cytoplasmic activity and having no significant impact on mitochondrial IDH2 and IDH3 in the tested conditions (Fig. 3E,F). Importantly, pharmacological blockade of IDH1 using GSK321 or AG‐120 abrogated the AR‐driven mitochondrial respiratory profile in LNCaP cells (Fig. 3G). Furthermore, IDH1 inhibition in VCaP cells significantly decreased this mitochondrial respiratory profile (Fig. 3H). These results were highly similar to those obtained using a genetic approach (*IDH1* knockdown; Fig. 3B,C). Moreover, a similar decrease in aerobic glycolysis, as measured using ECAR, was also observed following IDH1 inhibition (Fig. S3F,G). Altogether, these results demonstrate that IDH1 is critical to the bioenergetic profiles of PCa cells, notably supporting mitochondrial respiration of AR‐positive PCa cells.

### 3.4 Cytoplasmic IDH1 supports a hybrid cytoplasmic–mitochondrial TCA cycle

**Fig. 4:**
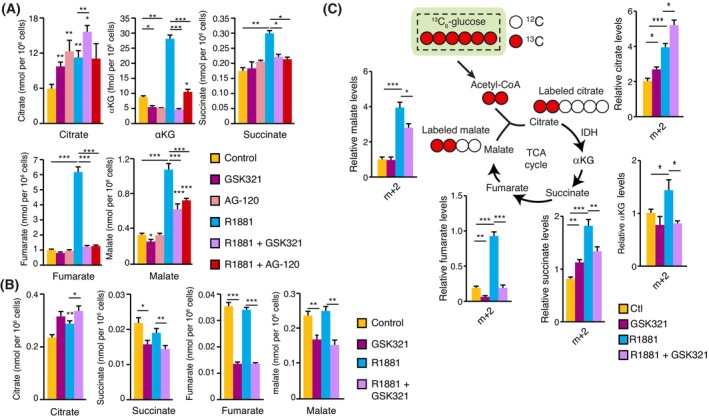
TCA cycle of PCa cell functions through an IDH1‐dependent hybrid cytoplasmic–mitochondrial pathway. (A) TCA cycle metabolite levels in LNCaP cells treated with or without the synthetic androgen R1881, with or without the IDH1 inhibitors GSK321 and AG‐120. GC–MS results are shown as the mean ± SEM (*n* = 5). (B) TCA cycle metabolite levels in 22Rv1 cells treated with or without R1881 and GSK321. GC–MS results are shown as the mean ± SEM (*n* = 4). (C) Stable isotope tracing analysis in LNCaP cells incubated for 24 h with ^13^C‐labelled glucose, treated with or without R1881 and GSK321. GC–MS results are shown as the mean ± SEM (*n* = 5). Statistics shown used the Student's *t*‐test. *P < 0.05; **P < 0.01; ***P < 0.001.

To understand how IDH1—a cytoplasmic enzyme—could be required for mitochondrial respiration, we resorted to targeted metabolomics using gas chromatography–mass spectrometry (GC–MS). In LNCaP cells, AR activation, which stimulates respiration (Fig. 3), also induced the levels of the different TCA cycle intermediates (Fig. S3H), as previously described [14, 15]. In these cells, inhibition of IDH1 did not block citrate synthesis following AR activation but rather increased citrate levels (Fig. 4A). However, all TCA cycle intermediate downstream of IDHs were significantly decreased following IDH1 inhibition, suggesting that it impaired the metabolic program controlled by AR (Fig. 4A), as predicted by mitochondrial respiration assays (Fig. 3). We then performed similar experiments with 22Rv1 cells, with and without R1881 and GSK321 (Fig. 4B). Androgens significantly increased citrate, while other metabolites remained at similar levels compared to control cells. Inhibition of IDH1, as seen in LNCaP cells, led to further accumulation of citrate while decreasing all the other detectable TCA cycle metabolites in 22Rv1 cells (Fig. 4B). Lastly, in VCaP cells, IDH1 inhibition also significantly altered TCA cycle intermediate levels, promoting the accumulation of citrate and further indicating an impaired TCA cycle following inhibition of IDH1 (Fig. S3I).

We then performed stable isotope tracer analysis using ^13^C‐labelled glucose to directly study TCA cycle activity in LNCaP cells. Carbon flux from glucose in all the detected intermediates from the TCA cycle significantly increased following AR activation (Fig. 4C). Upon co‐treatment with GSK321, carbon flux in citrate was further increased (Fig. 4C). However, IDH1 inhibition significantly impaired citrate usage, with a concomitant decrease in carbon flux through αKG, succinate, fumarate, and malate (Fig. 4C). Similar results were obtained using VCaP cells, also indicating decreased carbon flux in the TCA cycle following IDH1 inhibition (Fig. S3J). Altogether, these results indicate that cytoplasmic IDH1 is required for maximal mitochondrial TCA cycle and respiration in AR^+^ PCa cell lines.

### 3.5 IDH1 blockade impairs PCa cell proliferation *in vitro* and *in vivo*

**Fig. 5:**
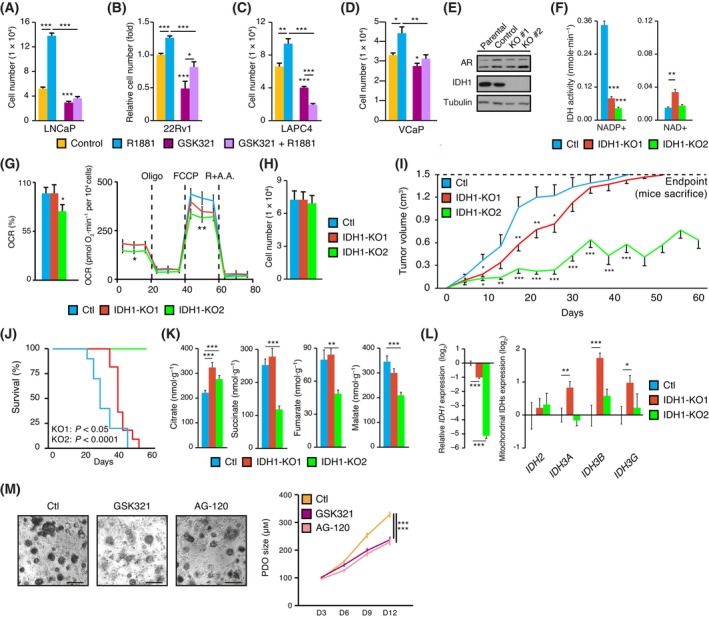
IDH1 represents a metabolic vulnerability in PCa. (A) LNCaP cell number after 7 days of treatment with or without the synthetic androgen R1881 and the IDH1 inhibitor GSK321. Results are shown as the mean ± SEM (*n* = 8). (B) 22Rv1 relative cell number after 7 days of treatment with or without R1881 and GSK321. Results are shown as the mean ± SEM (*n* = 8). (C) LAPC4 cell number after 6 days of treatment with or without R1881 and GSK321. Results are shown as the mean ± SEM (*n* = 8). (D) VCaP cell number after 6 days of treatment with or without R1881 and GSK321. Results are shown as the mean ± SEM (*n* = 4). For A–D, one representative independent experiment is shown out of at least three independent experiments. (E) IDH protein expression in IDH1‐KO 22Rv1 cells. Tubulin was blotted as a protein loading control. Parental and control 22Rv1 cells are shown along with two *IDH1* KO clones. One representative experiment is shown out of three independent experiments. (F) IDH activity associated with NADP^+^ and NAD^+^ in *IDH1* KO 22Rv1 cells compared to control cells (Ctl). Results are shown as the mean ± SEM (*n* = 3). (G) Basal respiration of *IDH1* KO 22Rv1 cells (left) and the complete mitochondrial stress test (right) are shown. Results are shown as the mean ± SEM (*n* = 10). In the complete mitochondrial stress panel, dashed lines indicate the sequential injections of oligomycin (oligo), the uncoupler FCCP, and rotenone + antimycin A (R + A.A.). (H) *IDH1* KO clone cell proliferation compared to control cells after 96 h of growth in complete media *in vitro*. Results are shown as the mean ± SEM (*n* = 8). (I) Tumour volumes of mice with xenografts from 22Rv1 control cells or *IDH1* KO cells. Mice were sacrificed when tumour volumes reached the endpoint (1.5 cm^3^). Results are shown as the mean ± SEM (*n* = 10–11/group). (J) Kaplan–Meier survival curves of mice with xenografts from 22Rv1 control cells or *IDH1* KO cells (*n* = 10–11/group). Mice were sacrificed when tumour volumes reached the endpoint (1.5 cm^3^). (K) TCA cycle metabolite levels in tumours of mice with xenografts from 22Rv1 control cells or *IDH1* KO cells. Results are shown as the mean ± SEM (*n* = 11–12/group). (L) IDH mRNA expression in tumours of mice with xenografts from 22Rv1 control cells or *IDH1* KO cells. Results are shown as the mean ± SEM (*n* = 8–9/group). (M) Brightfield visualization of human PCa patient‐derived organoids (PDOs) after 12 days of treatment with and without IDH1 inhibitors (left). On the right, quantification of the size of these PDOs treated with GSK321 or AG‐120. Scale bars = 250 μm. Analyses of survival rates were performed using the log‐rank statistical test; other statistics shown used the Student's *t*‐test. *P < 0.05; **P < 0.01; ***P < 0.001.

It is tempting to speculate that PCa cells bypass mitochondrial IDHs to induce mitochondrial respiration, despite the prostate‐specific truncated TCA cycle, by using their cytoplasmic counterpart IDH1. Consequently, PCa cells would rely on the existence of a hybrid cytoplasmic–mitochondrial TCA cycle that notably depends on IDH1. As such, given its key role in the AR‐dependent reprogramming of PCa cell metabolism, we hypothesized that blocking wild‐type IDH1 would also impair the proliferative capacity of PCa cells. First, we evaluated the effect of GSK321 on the proliferation of AR^+^ PCa cells (LNCaP, 22Rv1, LAPC4, and VCaP). As expected, AR activation induced a significant increase in proliferation in these cell lines (Fig. 5A–D). Pharmacological inhibition of IDH1 significantly decreased basal and AR‐induced proliferation (Fig. 5A–D). These results indicate that mutant IDH1 pharmacological inhibitors such as GSK321 could be repurposed to inhibit the proliferation of AR^+^ PCa cells even though they harbour wild‐type IDH1. Moreover, they also highlight wild‐type IDH1, and not only its mutated counterpart, as a potential therapeutic target for cancer treatment.

To further validate these results, we performed a CRISPR‐based knockout (KO) of *IDH1* in the 22Rv1 cell line. This specific cell line was chosen to allow clonal expansion, which was not possible with LNCaP or VCaP cells (data not shown). Several KO clones with deletions altering the open reading frame of *IDH1* were obtained (Fig. S4A–C), indicating an absence of IDH1 expression at the protein level (Fig. 5E). Indeed, both KO cell lines exhibited a loss of >70% of NADP^+^‐dependent IDH activity (Fig. 5F), consistent with the loss of IDH1 and the remaining activity being IDH2 dependent. Interestingly, IDH1‐KO1 cells, but not IDH1‐KO2 cells, exhibited a significant increase in the NAD^+^‐dependent IDH3 complex activity (Fig. 5F), possibly indicating a “rescue” of mitochondrial IDH activity to counteract the loss of IDH1. In line with these results, OCR analyses revealed similar respiration of IDH1‐KO1 compared to controls, while IDH1‐KO2 displayed an impaired respiratory profile with significantly lower basal and maximal respiration capacities (Fig. 5G). Moreover, the 20% increase in OCR observed in IDH1‐KO1 compared to IDH1‐KO2 was lost upon knockdown of *IDH3A*, reinforcing a possible IDH3‐dependent rescue mechanism in these IDH1‐KO1 cells (Fig. S4D,E). Consequently, distinct phenotypes similar to the emergence of treatment‐resistant mechanisms were observed in these IDH1‐KO clones. IDH1‐KO1 exhibited a rescue of mitochondrial respiration through increased IDH3 activity, while IDH1‐KO2 had no rescue of IDH activity, displaying decreased respiratory capacity.

Given the impact of knockdown or inhibition of IDH1 on cell metabolism and proliferation in acute assays (Figs 3, 4, and 5A–D), we performed proliferation assays in these *IDH1* KO clones. Surprisingly, we observed no effect of *IDH1* KO on the proliferation of these clones (Fig. 5H). We thus hypothesized that long‐term loss of *IDH1* in these cells led them to bypass—at least partially—their initial dependency on this enzyme. Nonetheless, this would imply that they have impaired metabolic flexibility potentially abrogating growth when the environment is less permissive than culture media, such as in physiological settings. Previous studies have shown a similar phenotype; the peroxisome proliferator‐activated receptor‐gamma coactivator‐1 alpha (PGC‐1α), for example, is a transcriptional cofactor that promotes mitochondrial biogenesis and activity. Its overexpression in a breast cancer model *in vitro* decreased proliferation despite significantly increasing mitochondrial content and respiratory capacity of cancer cells [44, 45]. However, in an *in vivo* context, the same cancer cells exhibited a reverse phenotype, leading to an increased growth advantage compared to control cells in this more restrictive environment [44, 45]. Given that loss of IDH1 is predicted to impair their metabolic flexibility, we next tested the growth of IDH1 KO clones *in vivo* in a xenograft assay. In this *in vivo* context, the two *IDH1* KO clones showed a significant delay in tumour growth and a longer time required to reach the xenograft study's endpoint compared to control cells (Fig. 5I,J), with loss of *IDH1* leading to increased survival. Tumours from IDH1‐KO2 were even incapable of reaching the endpoint, i.e. a tumour volume ≥ 1.5 cm^3^, and were thus sacrificed after all the mice from the other conditions reached the study endpoint (Fig. 5I).

This demonstrates that the growth of IDH1‐KO PCa cells is disrupted in an *in vivo* metabolic environment, correlating with a decrease in metabolic flexibility of these cancer cells. Intriguingly, despite both clones being significantly less aggressive in the xenograft assay, the IDH1‐KO2 displayed a much slower tumour growth rate compared to IDH1‐KO1, with an incapacity of these tumours to grow over a volume of 1 cm^3^ through the duration of the study (Fig. 5I). The severe blockade of IDH1‐KO2 growth *in vivo* correlated with their impaired IDH activity and mitochondrial respiration *in vitro* (Fig. 5F,G). Yet, IDH1‐KO1 cells remained significantly disadvantaged compared to control cells (Fig. 5I,J) despite having increased IDH3 activity as a rescue for the loss of IDH1 (Fig. 5F). These results show that loss of IDH1 leads to severe defects in tumours growth. Even in cases where cancer cells can adapt to the loss of IDH1 over time, such as restoring IDH3 complex activity, they nevertheless remain significantly disadvantaged compared to tumours exhibiting wild‐type IDH1 for growth *in vivo*, further emphasizing the importance of IDH1 in PCa cells. In xenograft tissues of *IDH1* KO cells (Fig. S4C), citrate was significantly higher after the loss of *IDH1* in both KO cell lines (Fig. 5K), as observed in cell lines that have undergone IDH1 inhibition (Fig. 4 and Fig. S3). Other detectable TCA cycle metabolites were succinate, fumarate, and malate. In IDH1‐KO2, correlating with a severely impaired growth, these metabolites were all significantly decreased compared to control tumours (Fig. 5K; additional metabolites related to the TCA cycle are shown in Fig. S4F). In IDH1‐KO1 cells, correlating with a partial rescue of IDH3 activity, similar levels of downstream intermediates compared to control tumours were observed (Fig. 5K). All three IDH3 genes (*IDH3A*, *IDH3B*, *IDH3G*) are required to form the IDH3 mitochondrial complex. At the mRNA level, they were all significantly higher in the IDH1‐KO1 tumours compared to control tumours, a phenomenon not observed in IDH1‐KO2 tumours (Fig. 5L).

Finally, we wanted to explore whether IDH1 inhibitors could be used to impair human PCa growth. As a proof‐of‐principle in a pilot study, we used one PCa PDO line and treated it with either vehicle, GSK321 or AG‐120 for 12 days. Under standard culture conditions, this PCa PDO grew to up to 300 μm in average after 12 days in culture (Fig. 5M). In contrast, PDOs that were treated with IDH1 inhibitors showed a significant decrease in growth, further reinforcing the hypothesis that IDH1 represents a metabolic vulnerability in PCa. Altogether these results show that both genetic and pharmacological blockades of IDH1 impair PCa proliferation and growth in *in vitro*, *in vivo*, and *ex vivo* preclinical models.

## 4 Discussion

**Fig. 6:**
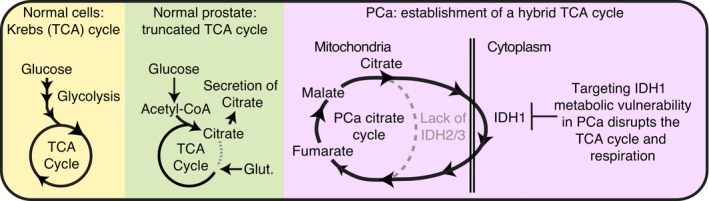
Prostate cancer cells are characterized by a hybrid cytoplasmic–mitochondrial TCA cycle that requires IDH1. Working model of how IDH1 contributes to the mitochondrial TCA cycle in PCa cells. In most eukaryotic cells, a canonical TCA cycle is observed. The prostate is metabolically unique as it produces and secretes massive amounts of citrate, an important function of the prostate gland that is required for fertility. To enable this unique metabolic phenotype, metabolites of the TCA cycle in prostate cells can be regenerated by different nutrients such as glucose through glucose oxidation, glutamine (glut) through glutaminolysis, and aspartate, using various entry points [5]. In PCa, citrate metabolism is reprogrammed to allow citrate usage for energy synthesis and mitochondrial respiration. However, a hybrid cytoplasmic‐mitochondrial TCA cycle is established through the high activity of IDH1. IDH2 activity and IDH3 activity remain low, as in the normal prostate, allowing targeting IDH1 as a PCa metabolic vulnerability. Note that this hybrid TCA cycle is most probably not exclusive and probably contributes, along with the canonical TCA cycle, to maximize bioenergetic potential of PCa cells.

Reprogramming of metabolism is now a well‐established hallmark of cancer [46]. Being specific to their lineage of origin or the metastatic niche to be invaded, metabolic adaptations allow tumour cells to rewire their nutrient consumption and alter their bioenergetics to enhance proliferative capabilities. Herein, we identified the IDH1 enzyme as an essential component of the AR‐driven metabolic program in PCa. Mechanistically, IDH1 supports a hybrid cytoplasmic–mitochondrial TCA cycle driven by AR in PCa cells. Our study highlights wild‐type IDH1 as a key enzyme required for PCa cell metabolism by causing an unusual metabolic adaptation to support mitochondrial respiration and uncovering a possible new therapeutic target for PCa (Fig. 6).

It is tempting to speculate that PCa cells bypass ACO2 and mitochondrial IDHs, by using their cytoplasmic counterpart IDH1, to induce mitochondrial respiration despite the prostate‐specific truncated TCA cycle. Consequently, a hybrid cytoplasmic–mitochondrial TCA cycle most probably requires additional enzymes, notably aconitase 1 (ACO1) upstream of IDH1 as well as specific mitochondrial transporters to allow shuttling of metabolites between subcellular compartments. The identification of these components remains to be fully determined. We believe that the establishment of this unusual cancer cell metabolic program originates from the unique prostate‐specific truncated TCA cycle, as recently studied by Frégeau‐Proulx et al. [5].

Mutant IDH1 has been extensively studied over the past decade and is an established clinical target in various brain and blood cancers. Indeed, studies report that when mutated, IDH1 will produce 2‐HG in sufficient amounts to competitively counteract αKG, a necessary cofactor for the activation of dioxygenase enzymes involved in genomic stability. Accumulation of 2‐HG thus disrupts the integrity of the genome and the epigenome, eventually amounting to oncogenesis [42, 43]. As *IDH1* is rarely mutated in PCa cells, we repurposed mutant IDH1 inhibitors in our models to induce a pharmacological blockade of IDH1. Concentrations used herein (1–5 μm) to inhibit PCa cells with wild‐type IDH1 correspond to the concentrations used to specifically kill acute myelogenous leukaemia (AML) cells with mutant IDH1 with no impact on AML cells with wild‐type IDH1 [38]. Thus, PCa cells seem more sensitive to wild‐type IDH1 inhibition than in other cancer cell types, possibly due to their specific lineage. We believe this is because PCa cells lack a functional mitochondrial IDH3 complex (Figs 1 and 2), the notion of which was also suggested by respiration assays in human PCa mitochondria [47], and because PCa exhibits a unique TCA cycle for which IDH1 is essential (Fig. 6). As PCa cells already exhibit low/absent mitochondrial IDH activity due to their prostatic lineage, further blockade of the cytoplasmic pathway completely abrogates total IDH activity and impairs proliferation and growth. The development of inhibitors specific to wild‐type IDH would facilitate their deployment to treat PCa.

## 5 Conclusions

In conclusion, the current study demonstrates that PCa cells exhibit a hybrid cytoplasmic–mitochondrial TCA cycle that depends on IDH1, an actionable target representing an important PCa metabolic vulnerability.

## Conflict of interest

The authors declare no conflict of interest.

## Author contributions

KG performed experiments, developed methods, analysed results, and wrote the manuscript. CW, LB, CJ, and MH performed experiments, developed methods, analysed results, helped write the manuscript and provided intellectual input. AL and CL performed experiments and provided intellectual input. BN and MS helped with method development and provided intellectual input. JL and AB provided intellectual input. YF, LL, JR, EL, CA, and FP performed surgery and tissue harvesting, helped with method development, and provided intellectual input. J‐PL and MP provided equipment and provided intellectual input. EA‐W conceived and supervised the project, performed experiments, analysed data, secured funding, and wrote the manuscript. All authors contributed to the manuscript and approved it for submission.

### Peer review

The peer review history for this article is available at https://www.webofscience.com/api/gateway/wos/peer‐review/10.1002/1878‐0261.13441.

## Supporting information


**Fig. S1.** IDH activity in PCa models in support of Fig. 1.
**Fig. S2.** IDH expression in mouse and human prostate models in support of Fig. 2.
**Fig. S3.** The TCA cycle of PCa cells functions through an IDH1‐mediated hybrid cytoplasmic‐mitochondrial pathway (in support of Figs 3 and 4).
**Fig. S4.** Establishment of a cell model genetically invalid for IDH1 in support of Fig. 5.
**Table S1.** Human qRT‐PCR primers.
**Table S2.** Human shRNA sequences.

## Data Availability

The data generated in this study are available upon request from the corresponding author.
