# Supplementary material for: Isocitrate dehydrogenase 1 sustains a hybrid cytoplasmic–mitochondrial tricarboxylic acid cycle that can be targeted for therapeutic purposes in prostate cancer
Source: Mol Oncol. 2023 Jul 19;17(10):2109–25. doi: 10.1002/1878-0261.13441 (PMC10552900; doi:10.1002/1878-0261.13441)
Supplement: Supplementary file 1 — Fig. S1. IDH activity in PCa models in support of Fig. 1. Fig. S2. IDH expression in mouse and human prostate models in support of Fig. 2. Fig. S3. The TCA cycle of PCa cells functions through an IDH1‐mediated hybrid cytoplasmic‐mitochondrial pathway (in support of Figs 3 and 4). Fig. S4. Establishment of a cell model genetically invalid for IDH1 in support of Fig. 5. Table S1. Human qRT‐PCR primers. Table S2. Human shRNA sequences. [file MOL2-17-2109-s001.docx]

***­Supplementary Material***

**Isocitrate dehydrogenase 1 sustains a hybrid cytoplasmic–mitochondrial tricarboxylic acid cycle that can be targeted for therapeutic purposes in prostate cancer**

Kevin Gonthier, Cindy Weidmann, Line Berthiaume, Cynthia Jobin, Aurélie Lacouture, Camille Lafront, Mario Harvey, Bertrand Neveu, Jérémy Loehr, Alain Bergeron, Yves Fradet, Louis Lacombe, Julie Riopel, Éva Latulippe, Chantal Atallah, Michael Shum, Jean-Philippe Lambert, Frédéric Pouliot, Martin Pelletier, and Étienne Audet-Walsh

**Supplementary Figures S1 – S4**

**Supplementary Tables S1 – S2**

**
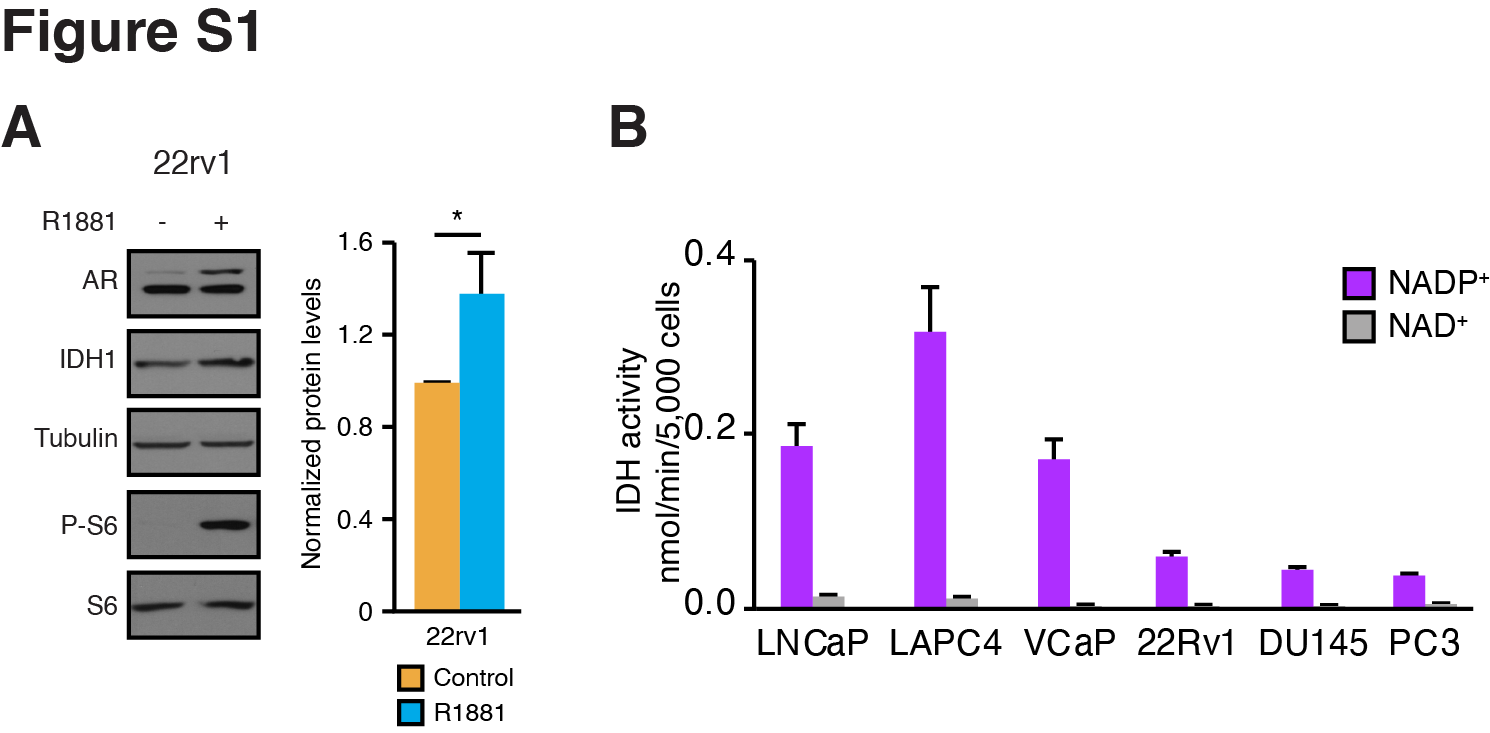
**

**Fig. S1. A)** IDH1 mRNA and protein expression levels in 22Rv1 cells following 48h of treatment with R1881 (10 nM). Tubulin was blotted as a protein loading control and phosphorylation of S6 (P-S6) is used as a positive control for AR activation. Densitometric quantification of IDH1 intensity over tubulin intensity is shown as the mean of three independent experiments. Total IDH activity fold change following treatment with R1881 (10 nM) is shown as mean ± SEM (*n* = 3). **B)** NADP^+^-dependent and NAD^+^-dependent IDH activity in multiple PCa cell lines (AR^+^; LNCaP, LAPC4, VCaP, 22Rv1: AR^-^; DU145, PC3). Results are shown as mean ± SEM (*n* = 3). Statistics shown used the Student’s *t*-test. *: *P*<0.05.


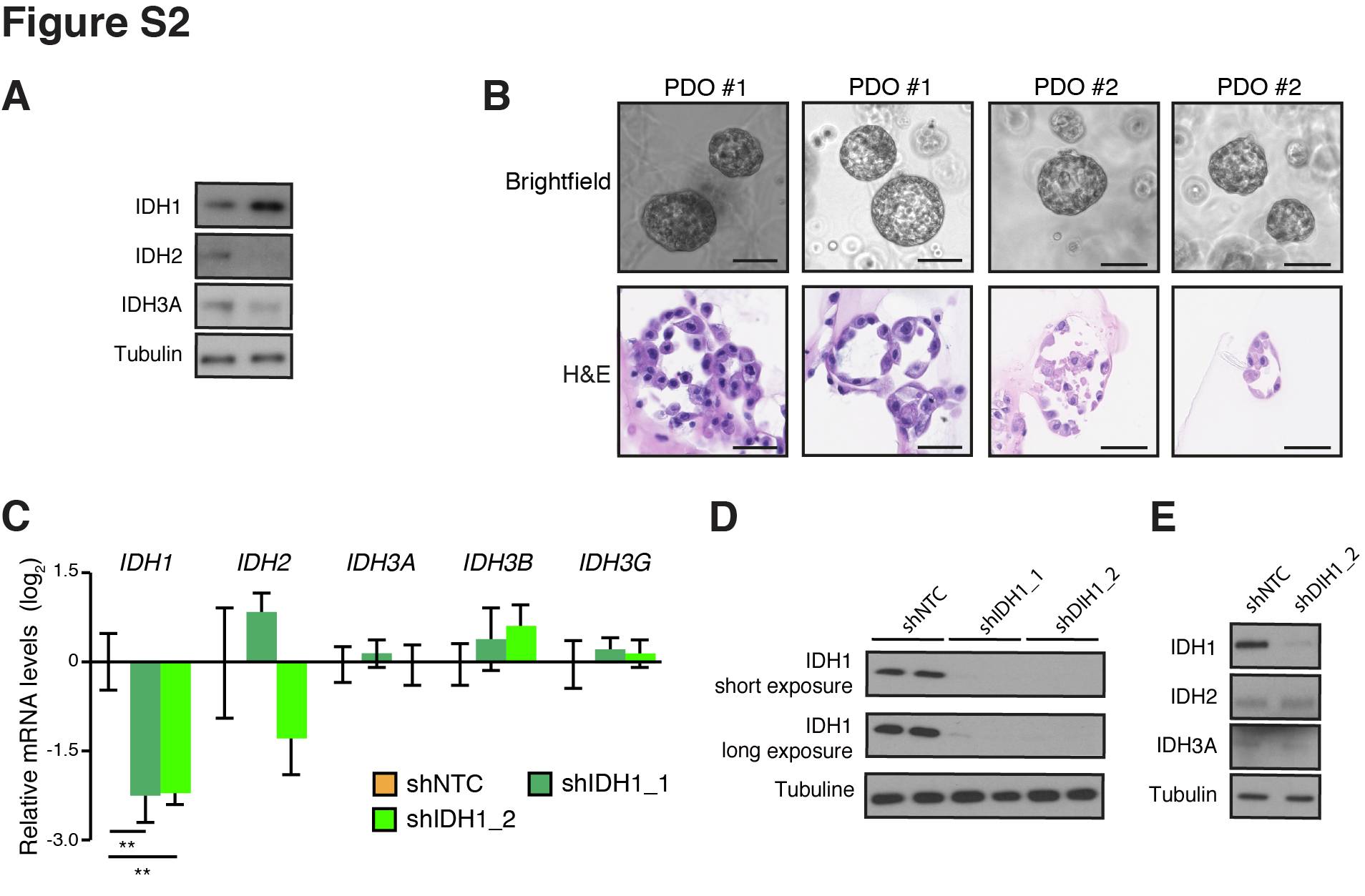


**Fig. S2.** **A)** IDH protein expression levels in two mouse prostate tissues (whole cell lysate samples). IDH2 and IDH3A levels were close to our detection limit. Tubulin is shown as a loading control. **B)** Brightfield visualization of human prostate organoids after 14 days in culture. Scale bars = 100 µm. Hematoxylin & eosin (H&E) staining is also shown to indicate internal lumen, as expected for a glandular structure. bars = 50 µm. **C)** qRT-PCR analysis of IDHs genes expression in LNCaP cells. Results are shown as the mean ± SEM (*n* = 3) of one out of two independent experiments. IDH1 protein expression in LNCaP (**D**) and PDO (**E**) cells following expression of shIDH1. Tubulin is shown as a loading control. In E, note that IDH2 and IDH3A protein levels were closed to our detection limit. Statistics shown used the Student’s *t*-test. *: *P*<0.05; **: *P*<0.01; ***: *P*<0.001.

**
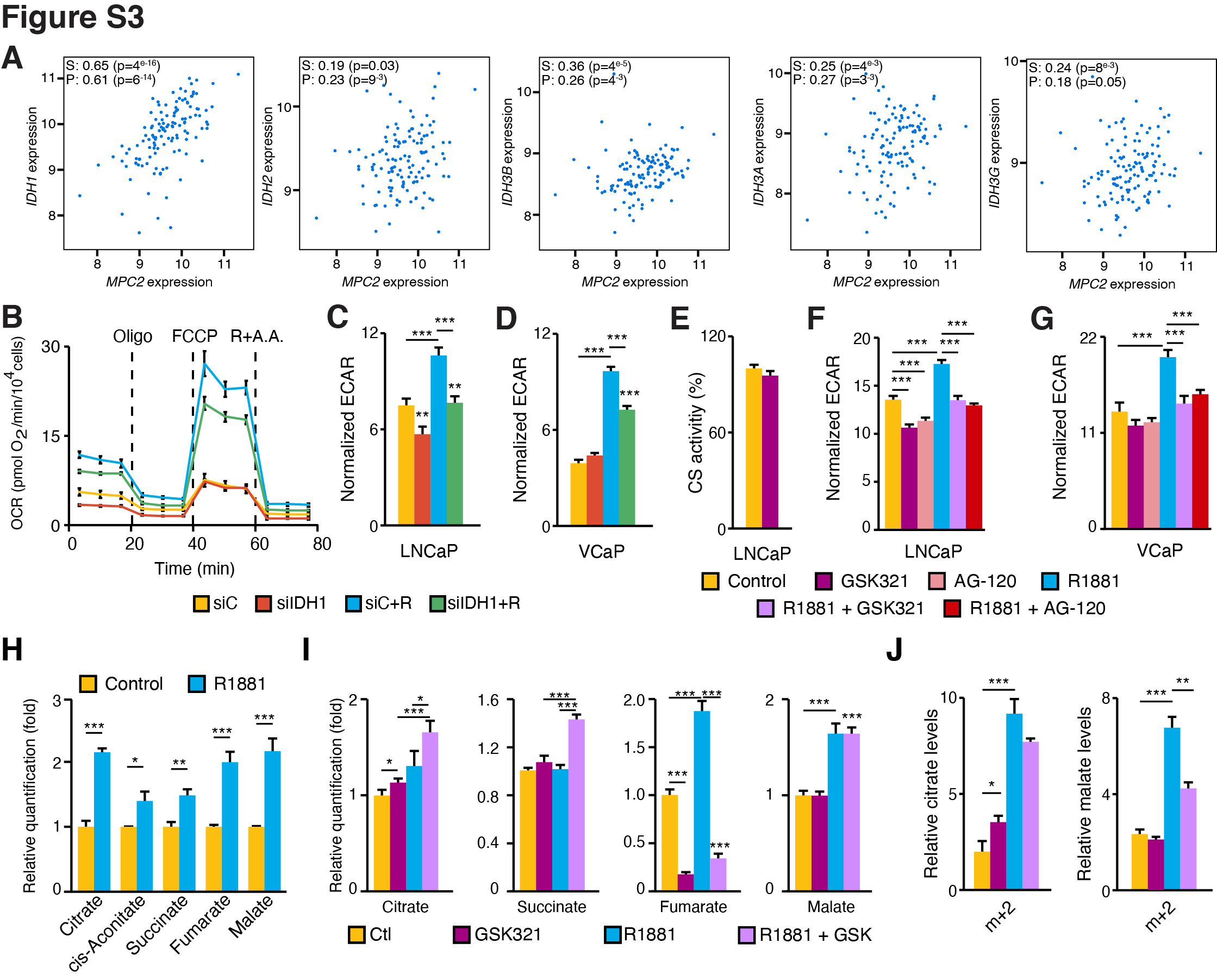
**

**Fig. S3.** TCA cycle of PCa cells functions through an IDH1-mediated hybrid cytoplasmic-mitochondrial pathway. **A)** Correlation analysis of *IDH1*, *IDH2*, *IDH3A*, *IDH3B* and *IDH3G* mRNAs with *MPC2* mRNA in the Taylor *et al.* dataset. **B)** Oxygen consumption rates (OCR) of VCaP cells treated with vehicle or R1881 (10 nM) for 48h and transfected with scrambled-siRNA (siC) or IDH1-targeting siRNA (siIDH1) for 96h. OCR in a mitochondrial stress test is shown and results are shown as mean ± SEM (*n* = 10–12). Extracellular acidification rates (ECAR) of LNCaP (**C**) and VCaP (**D**) cells treated with vehicle or R1881 (10 nM) for 48h and transfected with scrambled-siRNA (siC) or IDH1-targeting siRNA (siIDH1) for 96h. **E)** Citrate synthase (CS) activity with and without 48h treatment with the IDH1 inhibitor GSK321 in LNCaP cells. Results are shown as the average and SEM of two independent experiments performed in quadruplicates. ECAR of LNCaP (**F**) and VCaP (**G**) cells treated with vehicle, R1881, GSK321 or R1881+GSK321 for 48h. For C-F, results of one representative experiment out of 3 independent experiments are shown. **H)** Fold increase of TCA cycle metabolites levels in LNCaP cells treated after treatment with R1881 (10 nM) for 48h. Results are shown as mean ± SEM (*n* = 4). **I)** Relative TCA cycle metabolite levels in VCaP cells treated for 48h with or without R1881 (10 nM) and treated or not with IDH1 inhibitor GSK321 (5 µM). GC-MS results are shown as the average of two independent experiments ± SEM (*n* = 4). **J)** Stable isotope tracing analysis in VCaP cells incubated for 24h with ^13^C-labelled glucose, treated with or without R1881 (10 nM) or GSK321 (5 µM). Results are shown as mean ± SEM (*n* = 5) for citrate and malate. Other metabolites were undetectable. For malate, m+4 was undetectable. Statistics shown used the Student’s *t*-test. *: *P*<0.05; **: *P*<0.01; ***: *P*<0.001.

**
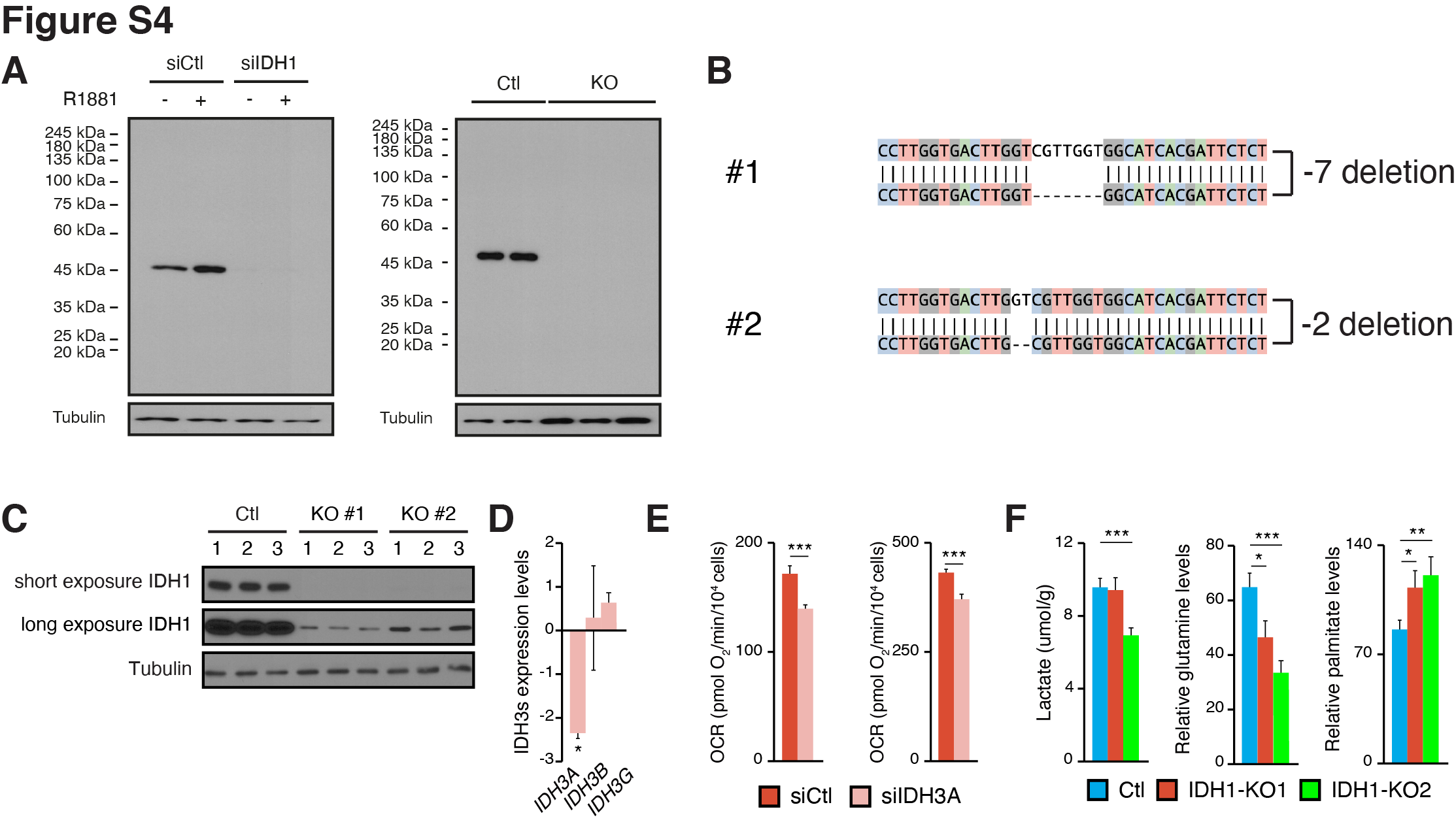
Fig. S4.** Establishment of a cell model genetically invalid for IDH1. **A)** Specificity of IDH1 antibody (ab172964) is validated in LNCaP cells transfected with a scrambled siRNA or IDH1-targeting siRNA, with and without R1881 treatment (left), and in 22Rv1 control (ctl) cells or 22Rv1 IDH1-KO cells (right) using a western blot analysis. Tubulin was blotted as a loading control. **B)** Confirmation of CRISPR-mediated sequence disruption of IDH1-exon 4 using Sanger sequencing. **C)** IDH protein expression in tumors of mice with xenografts from 22Rv1 control cells or *IDH1* KO cells. Tubulin was blotted as a protein loading control. **D)** Specific *IDH3A* knockdown following transfection with siRNA targeting IDH3A (siIDH3A) in IDH1-KO1 cells (expression levels are shown in log_2_). IHD3 gene expression levels in cells transfected with control siRNA are set at 0. Results are shown as the average and SEM of two independent experiments. **E**) Oxygen consumption rates (OCR) of IDH1-KO1 cells transfected with scrambled-siRNA (siC) or IDH3A-targeting siRNA (siIDH3A) for 72h. Basal and maximal OCR are shown as average and SEM of one experiment out of four independent experiments. **F)** TCA cycle metabolite levels in tumors of mice with xenografts from 22Rv1 control cells or *IDH1* KO cells. Results are shown as mean ± SEM (*n* = 11–12/group). Statistics shown used the Student’s *t*-test. *: *P*<0.05; **: *P*<0.01; ***: *P*<0.001.

**Supplementary Tables**

**Supplementary Table 1. Human qRT-PCR primers**

| Primer | Sequence | Organism |
| --- | --- | --- |
| *TBP* forward | TGCCACGCCAGCTTCGGAGA | *Homo sapiens* |
| *TBP* reverse | ACCGCAGCAAACCGCTTGGG | *Homo sapiens* |
| *PUM1* forward | ACGGATTCGAGGCCACGTCC | *Homo sapiens* |
| *PUM1* reverse | CATTAATTACCTGCTGGTCTGAAGGA | *Homo sapiens* |
| *IDH1* forward | CAGGCTGTGGTTGTGAGTCT | *Homo sapiens* |
| *IDH1* reverse | TAGTTTATCGCCTGCCGGG | *Homo sapiens* |
| *IDH2* forward | GCTCTCCAGCTTGGGATGG | *Homo sapiens* |
| *IDH2* reverse | GCTTCGCCACCTTGATCCTT | *Homo sapiens* |
| *IDH3A* forward | ACTGGTGGTGTTCAGACAGT | *Homo sapiens* |
| *IDH3A* reverse | TGAATGGCAGTGACGTTCCG | *Homo sapiens* |
| *IDH3B* forward | ATCAAAGTTGGCAAGGTGCG | *Homo sapiens* |
| *IDH3B* reverse | GAGGCACAAGGTCTCTTCCC | *Homo sapiens* |
| *IDH3G* forward | AACAGCTACGAGGAACACCG | *Homo sapiens* |
| *IDH3G* reverse | CGGAGTGTGCATATTCTCATTGT | *Homo sapiens* |

**Supplementary Table 2. Human shRNA sequences**

| Primer | Sequence | Organism |
| --- | --- | --- |
| shNTC forward | CTAGCCAACAAGATGAAGAGCACCAATACTAGTTTGGTGCTCTTCATCTTGTTGTTTTTG | *Homo sapiens* |
| *shNTC* reverse | AATTCAAAAACAACAAGATGAAGAGCACCAAACTAGTATTGGTGCTCTTCATCTTGTTGG | *Homo sapiens* |
| *shIDH1_1* forward | CTAGCCGAATCATTTGGGAATTGATTTACTAGTAATCAATTCCCAAATGATTCGTTTTTG | *Homo sapiens* |
| *shIDH1_1* reverse | AATTCAAAAACGAATCATTTGGGAATTGATTACTAGTAAATCAATTCCCAAATGATTCGG | *Homo sapiens* |
| *shIDH1_2* forward | CTAGCGCTGCTTGCATTAAAGGTTTATACTAGTTAAACCTTTAATGCAAGCAGCTTTTTG | *Homo sapiens* |
| *shIDH1_2* reverse | AATTCAAAAAGCTGCTTGCATTAAAGGTTTAACTAGTATAAACCTTTAATGCAAGCAGCG | *Homo sapiens* |
